# Attitudes of Australians with inflammatory arthritis to biologic therapy and biosimilars

**DOI:** 10.1093/rap/rkac099

**Published:** 2022-11-10

**Authors:** Alannah Quinlivan, Susan Lester, Claire Barrett, Samuel Whittle, Debra Rowett, Rachel Black, Vibhasha Chand, Franca Marine, Lyn March, Premarani Sinnathurai, Rachelle Buchbinder, Catherine Hill

**Affiliations:** Rheumatology Unit, Queen Elizabeth Hospital, Woodville, SA, Australia; Rheumatology Unit, Queen Elizabeth Hospital, Woodville, SA, Australia; University of Adelaide, Adelaide, SA, Australia; Redcliffe Hospital, Redcliffe, QLD, Australia; Discipline of Medicine, University of Queensland, Brisbane, QLD, Australia; Rheumatology Unit, Queen Elizabeth Hospital, Woodville, SA, Australia; Department of Epidemiology and Preventive Medicine, School of Public Health and Preventive Medicine, Monash University, Brisbane, VIC, Australia; Monash-Cabrini Department of Musculoskeletal Health and Clinical Epidemiology, Cabrini Health, Malvern, VIC, Australia; UniSA Clinical and Health Science, University of South Australia, Adelaide, SA, Australia; Drug and Therapeutics Information Service, Southern Adelaide Local Health Network, Daw Park, SA, Australia; Rheumatology Unit, Queen Elizabeth Hospital, Woodville, SA, Australia; University of Adelaide, Adelaide, SA, Australia; Rheumatology Unit, Royal Adelaide Hospital, Adelaide, SA, Australia; Department of Epidemiology and Preventive Medicine, School of Public Health and Preventive Medicine, Monash University, Brisbane, VIC, Australia; Monash-Cabrini Department of Musculoskeletal Health and Clinical Epidemiology, Cabrini Health, Malvern, VIC, Australia; Arthritis Australia, Sydney, NSW, Australia; Institute of Bone and Joint Research, University of Sydney, Sydney, NSW, Australia; Department of Rheumatology, Royal North Shore Hospital, St Leonards, NSW, Australia; Institute of Bone and Joint Research, University of Sydney, Sydney, NSW, Australia; Department of Rheumatology, Royal North Shore Hospital, St Leonards, NSW, Australia; NPS MedicineWise, Sydney, NSW, Australia; Department of Epidemiology and Preventive Medicine, School of Public Health and Preventive Medicine, Monash University, Brisbane, VIC, Australia; Monash-Cabrini Department of Musculoskeletal Health and Clinical Epidemiology, Cabrini Health, Malvern, VIC, Australia; Rheumatology Unit, Queen Elizabeth Hospital, Woodville, SA, Australia; University of Adelaide, Adelaide, SA, Australia; Rheumatology Unit, Royal Adelaide Hospital, Adelaide, SA, Australia

**Keywords:** patient attitude towards health, biologic therapies, biosimilars, RA, axial spondyloarthritis, PsA, inflammatory arthritis

## Abstract

**Objectives:**

To investigate the knowledge and beliefs of Australian patients with inflammatory arthritis regarding biologic/targeted synthetic DMARDs (b/tsDMARDs) and biosimilars and their sources of information.

**Methods:**

Participants enrolled in the Australian Rheumatology Association Database (ARAD) with RA, PsA and axial SpA were sent an online survey. They were asked about information sources for b/tsDMARDs and how positive or negative this information was. The Beliefs about Medicine Questionnaire (BMQ) was used to measure beliefs about b/tsDMARDs with scores ranging from 1 (strongly disagree) to 5 (strongly agree). Participants were asked about their knowledge of biosimilars and willingness to switch to biosimilar.

**Results:**

There was a response rate of 66% (994/1498; 67% female, median age 62 years). Participants currently taking b/tsDMARDs (*n* = 794) had a high b/tsDMARD-specific BMQ ‘necessity’ score {median 4.2 [interquartile range (IQR) 3.6–4.8]}, with a lower specific ‘concerns’ score [median 2.4 (IQR 2.0– 3.0)]. Participants consulted multiple information sources [median 3 (IQR 2–5)]. Positive sources were rheumatologists and educational websites and negative were chat rooms and social media. Only 18% were familiar with biosimilars, with half knowing of availability in Australia. Following a short paragraph describing biosimilars, 75% (744) of participants indicated they would consider switching if recommended by their rheumatologist, with nearly half identifying safety and efficacy of biosimilars as an important concern.

**Conclusion:**

Australian patients have positive attitudes towards b/tsDMARDs overall, although little knowledge of biosimilars specifically. They have a high degree of trust in their rheumatologist regarding treatment decisions, even if they are unfamiliar with the medication recommended.

Key messages

- Australian patients with RA, PsA and axial SpA have positive attitudes towards b/tsDMARDs overall, although little knowledge of biosimilars specifically.
- Australian patients have a high degree of trust in their rheumatologist, even if unfamiliar with the medication recommended.

## Introduction

RA, PsA and axial SpA (axSpA) are systemic, autoimmune inflammatory conditions that can result in debilitating joint pain and deformities if disease control is not obtained. In 2020, data from the Global Burden of Disease Study reported that musculoskeletal conditions, including RA, PsA and axSpA, are the second greatest cause of disability worldwide [1]. The introduction of biologic/targeted synthetic DMARDs (b/tsDMARDs) has improved disease control and resulted in disease remission or low disease activity in many patients with rheumatic disease previously uncontrolled with conventional synthetic DMARDs (csDMARDs) alone. While the use of these agents is becoming increasingly common worldwide, their cost remains an issue, especially in lower-income countries [2]. Encouraging the uptake of biosimilars, which are biological products highly similar to original b/tsDMARDs but 20–40% less expensive [3], may enable improved access to biologic treatments for patients with inflammatory arthritis.

In Australia, the federal government subsidizes medicines found to be cost effective to make them more affordable to the general population through the Pharmaceutical Benefits Scheme (PBS). In Australia, b/tsDMARDs accounted for 8 of the top 10 PBS-subsidised drugs by expenditure in 2018–2019 [4]. The potential cost savings of successful introduction of biosimilars are significant, with a study undertaken in the UK demonstrating cost savings of >£38 million over 2 years due to the introduction of only two of the available biosimilars (etanercept and infliximab biosimilars) [5].

Biosimilars have been available in Australia in the last 5 years for some b/tsDMARDs (i.e. infliximab, rituximab, etanercept and adalimumab). In 2015, the Australian government introduced the Biosimilar Awareness Initiative, a program to increase awareness of biosimilars among medical professionals and patients. However, the success of the program to increase awareness of biosimilars among patients is unknown.

Patients will generally seek out health information from a variety of sources to aid them in reaching decisions about their health [6–8]. Numerous studies have demonstrated a link between patients’ beliefs regarding their medication and medication adherence [9]. Therefore the quality and positivity of the information patients receive regarding b/tsDMARDs and biosimilars has the potential to influence their beliefs about these medications and their willingness to adhere to treatment [10–12]. The primary aims of this study were to investigate the knowledge and beliefs of Australian patients with inflammatory arthritis regarding b/tsDMARDs and their sources of information, as well as their knowledge about biosimilars and which factors would influence their decision to switch to a biosimilar.

## Methods

Participants in this study were recruited from the Australian Rheumatology Association Database (ARAD; www.arad.org.au). The ARAD is a national database that collects health information from individuals with inflammatory arthritis, including juvenile idiopathic arthritis. Participants can be referred to the ARAD by rheumatologists or via self-referral. Each participant gives written informed consent to be part of the ARAD and completes self-administered surveys at 6–12 month intervals.

A link to the study survey was sent via e-mail to ARAD participants with RA, PsA or axSpA who had completed an ARAD online questionnaire within the previous 12 months.

Study data were collected and managed from an online survey conducted in January 2020 using Research Electronic Data Capture (REDCap) hosted at the University of Sydney [13]. REDCap is a secure, web-based application designed to support data capture for research studies.

Survey responses were linked to demographic data in the ARAD, including diagnosis, sex, current age and education. Education level was defined as high school or less (secondary education), university, vocational diploma or certificate (tertiary education).

The survey tool is reported in Supplementary File 1, available at *Rheumatology Advances in Practice* online. All survey participants completed the general medicine Beliefs about Medicine Questionnaire (BMQ) [14], the Single Item Literacy Screener (SILS) [15] and current medication use for both cs- and b/tsDMARDs. Survey participants currently using b/tsDMARDs also completed the b/tsDMARD-specific BMQ (necessity and concerns) scales, and, in addition, were asked about the information sources they had utilized regarding these medications. Finally, all survey participants were asked a series of questions about their knowledge, familiarity and attitudes towards biosimilars.

The BMQ consists of general medication (overuse and harm) and b/tsDMARD-specific (necessity and concerns) questions. Responses to each question comprise a 5-point Likert scale ranging from 1 (strongly disagree) to 5 (strongly agree). The BMQ was designed for use in chronic disease [16] and has previously been used in trials of RA [14]. The SILS was used to screen for limited reading ability, which is one component of health literacy. Participants were asked how often they needed assistance reading health information materials, with response options ranging from 1 (always) to 5 (never). Scores of 1 (always) and 2 (often) are considered to indicate some difficulty with reading printed health-related material. When compared with the Short Test of Functional Health Literacy in Adults, the SILS had a sensitivity of 54% and a specificity of 83% [15].

Survey participants currently taking b/tsDMARDs were asked about different information sources they may have utilized regarding their medication (question 1.4, Supplementary File 1, available at *Rheumatology Advances in Practice* online). Potential sources were listed as rheumatologist, rheumatology nurse, general practitioner (GP), pharmacist, relative/friend, other patients, educational websites (such as the Australian Rheumatology Association or Arthritis Australia), other websites, social media (Facebook, Twitter, Instagram), chat rooms and other media (newspaper, magazines, television, radio). For each nominated source, survey participants were asked to rate how favourable this resource was on a seven-category ordinal scale ranging from very strongly negative to very strongly positive.

Knowledge and attitudes about biosimilars were addressed in sections 3 and 4 of the survey (Supplementary File 1, available at *Rheumatology Advances in Practice* online). Survey participants were specifically asked what would encourage them to switch from a b/tsDMARD to a biosimilar. Response options included medication properties (such as cost and method of administration), recommendation from a rheumatologists and proven safety/efficacy in clinical trials.

### Data analysis

Data analyses and tabulations were performed in Stata version 16 (StataCorp, College Station, TX, USA).

BMQ-harms and BMQ-overuse scores were calculated by summing participant scores within each of the harms and overuse domains in order to calculate a mean score (ranging from 1 to 5), as previously described [17]. Higher BMQ-harms or BMQ-overuse scores indicate that medications are perceived to be more harmful or overused, respectively.

The combinations of b/tsDMARD data sources (Supplementary File 1, question 1.4, available at *Rheumatology Advances in Practice* online) utilized by study participants were visualized in an upset chart, using the R library UpSetR (R Foundation for Statistical Computing, Vienna, Austria) [18, 19].

Exploratory analyses of the relationship between selected covariates and both b/tsDMARD information sources and their reported views were performed using a Heckman ordinal probit selection model (heckoprobit command in Stata) for each information source. These models estimate simultaneously the probability of an information source being selected (binary probit model) and, if selected, the perceived favourability of the information received (with the ordinal probit model for the seven ordered categories ranging from extremely negative to extremely positive). Covariates included in these analyses were age, gender, education, self-reported perceived reading ability (SILS) and b/tsDMARD-specific BMQ-harms and BMQ-overuse scores.

### Ethics/permissions

The ARAD has ethics approval from Monash University and other sites, including the Central Adelaide Local Health Network. Permission was obtained through the ARAD steering committee and the Central Adelaide Local Health Network Human Research Ethics Committee (reference number 12423; 14 January 2020) to conduct this survey.

## Results

### Survey participants

Of 1498 invited ARAD participants, 994 completed the survey (response rate 66%), comprising 648 respondents with RA, 180 with axSpA and 166 with PsA. The majority of participants were female [666 (67%)] and the median age was 63 years (Table 1). When compared with non-responders, survey responders were comparable by sex (67% *vs* 65% female) and diagnosis (65% *vs* 64% RA), but were slightly older (62 *vs* 59 years; *P* < 0.001).

**Table 1.** Demographics of survey participants, including the subgroup of participants currently taking b/tsDMARDs

Characteristics	All participants	b/tsDMARD subgroup
Patients, *n*	994	794
Rheumatic diseases, *n* (%)		
RA	648 (65)	519 (65)
axSpA	180 (18)	152 (19)
PsA	166 (17)	123 (15)
Females, *n* (%)	666 (67)	525 (66)
Age, years, median (IQR)	63 (55–70)	62 (53–69)
Age at diagnosis, years, median (IQR)	40 (30–52)	39 (29–50)
Education, *n* (%)		
University, diploma/certificate	644 (65)	518 (65)
Reading help required^a^, *n* (%)		
Always/often	20 (2)	13 (2)
Sometimes	49 (5)	38 (5)
Rarely/Never	925 (93)	744 (94)
Current medications, *n* (%)		
Methotrexate	495 (50)	395 (50)
Leflunomide	85 (9)	60 (8)
Prednisolone	238 (24)	182 (23)
b/tsDMARD	794 (80)	794 (100)

a SILS [15].

### DMARD use

Of the 994 survey participants, 794 (80%) were currently taking a b/tsDMARD, with 518/794 (65%) on their first b/tsDMARD. The demographics of this subset of survey participants are also reported in Table 1.

Of the 200 participants who were not currently taking a b/tsDMARD, 88 (44%) had previously been treated with a b/tsDMARD (with the most common reason for cessation being loss of efficacy), 9 (5%) had been offered a b/tsDMARD but had declined and 51 (26%) had never heard of b/tsDMARDs.

With regards to csDMARDs, 495/994 (50%) were currently taking methotrexate [436/495 (88%), oral administration], 85/994 (9%) were currently taking leflunomide and 238/994 (24%) were currently taking prednisolone (median dose of 5 mg/day).

### b/tsDMARD-specific beliefs about medicine

The b/tsDMARD-specific BMQ ‘necessity’ score was high in survey participants currently taking a b/tsDMARD (median 4.2 of a possible maximum score of 5; Table 2), indicating a strong belief in the necessity of their medication. In contrast, the median b/tsDMARD-specific ‘concerns’ score was relatively low (2.4; Table 2), and similar to both ‘overuse’ and ‘harms’ general medicine BMQ scores (also reported in Table 2), suggesting these participants had no greater concerns about b/tsDMARDs than medications in general. For participants who had previously used a b/tsDMARD but were not taking one currently, there was no relationship between the b/tsDMARD-specific BMQ score and the number of previous b/tsDMARDs they had been treated with. There were also no differences between the type of inflammatory arthritis and b/tsDMARD-specific BMQ scores.

**Table 2.** BMQ [16] scores for survey participants currently taking b/tsDMARDs (*n* = 794)

BMQ scale^a^	Median (IQR)
BMQ b/tsDMARD-specific: necessity	4.2 (3.6–4.8)
BMQ b/tsDMARD-specific: concerns	2.4 (2.0–3.0)
BMQ general medicine: harms	2.2 (1.8–2.6)
BMQ general medicine: overuse	2.7 (2.0–3.0)

a Range 1–5.

### b/tsDMARD information sources

**Figure 1.**
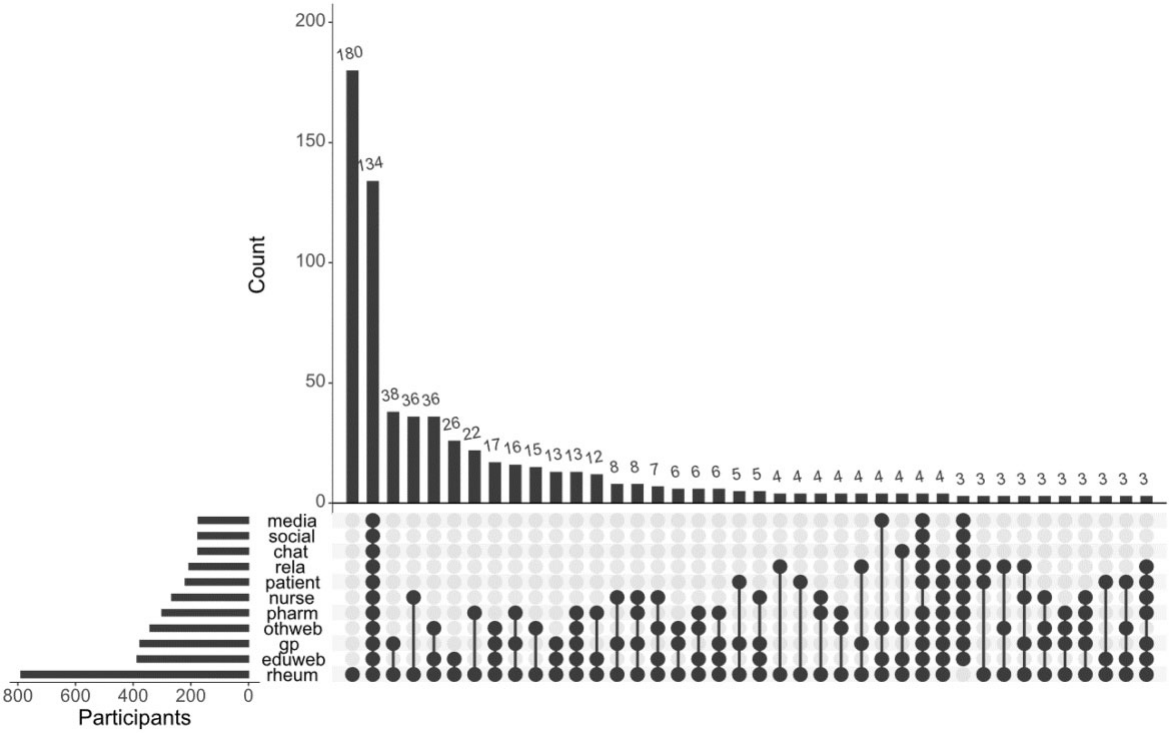
UpSet chart of the patterns and frequencies of reported b/tsDMARD information sources utilized by survey participants currently taking b/tsDMARDs (*n* = 794). Each pattern of dots shows each unique combination of information sources utilized and the histogram at the top shows the frequency/counts of each unique combination. The small histogram at the bottom left shows the counts for each information source

Survey participants were questioned about specific sources used for information on b/tsDMARDs and the positivity of the information they received (question 1.4, Supplementary File 1, available at *Rheumatology Advances in Practice* online). Survey participants currently taking b/tsDMARDs reported consulting multiple b/tsDMARD information sources {median 3 [interquartile range (IQR) 2–5]} in different combinations (Fig. 1).

**Figure 2.**
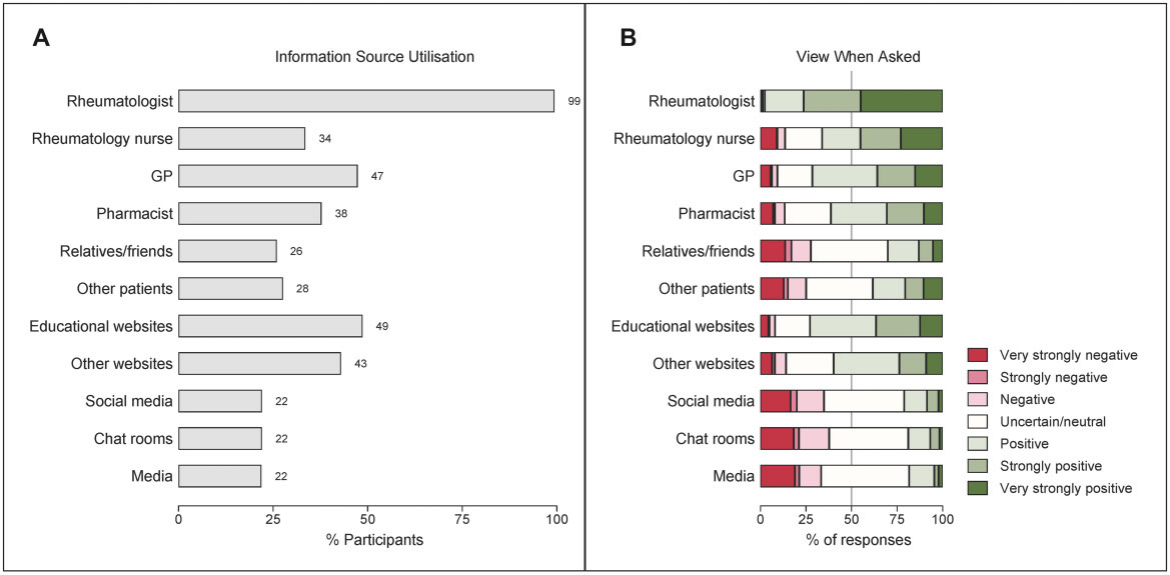
b/tsDMARD information obtained by survey participants currently on b/tsDMARD treatment (*n* = 794). (**A**) The proportion of survey participants who reported using specified information sources. (**B**) The distribution of the perceived information/views obtained, ranging from very strongly negative to very strongly positive

The utilization of b/tsDMARD information sources is presented in Fig. 2A. The most frequently reported b/tsDMARD information sources were rheumatologists [789 (99%)], followed by educational websites [386 (49%)], GPs [376 (47%)], ‘other’ websites such as Google or Wikipedia [341 (44%)], pharmacists [300 (38%)] and rheumatology nurses [266 (33%)]. Chat rooms/social media (such as Facebook, Twitter, Instagram), other media, relatives/friends and other patients were information sources reported by 175 (22%), 174 (22%), 206 (26%) and 219 (28%) participants, respectively. All but five survey participants reported their rheumatologist as an information source, and although a substantial proportion [180/789 (23%)] utilized only their rheumatologist, the majority [609/789 (77%)] also sought information from additional sources.

As expected, there was a wide range in how the b/tsDMARD information obtained was perceived (Fig. 2B), with nearly all the information obtained from rheumatologists perceived as positive to some extent. Other sources of mostly positive information included educational websites [281/286 (73% positive)], GPs [269/376 (71% positive)], rheumatology nurses [176/266 (66% positive)], pharmacists [184/300 (61% positive)] and other websites [204/342 (60% positive)]. Uncertainty, or a neutral perception, was the predominant response reported for other media [84/174 (48%)], social media [77/175 (44%)], chat rooms [76/175 (43%)] and relatives/friends [87/206 (42%)].

Despite the variability in the b/tsDMARD information received, overall satisfaction with the information obtained was high. The median satisfaction score, as measured on a 0–100 Likert scale, was 83 (IQR 70–94). Higher BMQ b/tsDMARD-specific necessity scores were correlated with higher satisfaction scores [Spearman’s ρ 0.24 (95% CI 0.17, 0.30)], whereas higher BMQ b/tsDMARD-specific concerns scores were correlated with lower satisfaction scores [Spearman’s ρ −0.43 (95% CI −0.49, −0.37)].

An exploratory analysis of the relationship between covariates [age, sex, education, self-reported perceived reading ability (SILS) and b/tsDMARD-specific BMQ-harms and BMQ-overuse scores] and both the selection of b/tsDMARD information sources and their associated views are reported in Supplementary Table S1, available at *Rheumatology Advances in Practice* online. While the analysis involves many comparisons, some inferences are of interest. First, younger patients were more likely to report views from a range of information sources and more likely to report positive views from internet sites. Second, patients with poorer health literacy were more likely to report consulting GPs and pharmacists. Third, higher BMQ b/tsDMARD-specific concerns may drive the range of sources consulted (e.g. relatives/friends, other websites, social media, chat rooms), whereas higher BMQ b/tsDMARD-specific necessity scores were associated with more favourable views reported for rheumatologists and GPs.

### Factors influencing the choice of b/tsDMARD

Survey participants currently taking b/tsDMARDs were also asked to select the most important factor (from a range of alternatives) influencing their choice of b/tsDMARD, assuming equal effectiveness. A rheumatologists recommendation was the most frequent response [559/794 (70%)], followed by cost to the patient [96 (12%)] and method of drug delivery [87 (11%)].

### Knowledge and perceptions of biosimilars

Of the 994 study participants, only 180 (18%) indicated they were familiar with biosimilars, with a further 67 (7%) unsure. Of those participants who stated they were familiar with the term ‘biosimilar’ or unsure, only 50% (124) were able to correctly define the term. A total of 51 participants (21%) believed biosimilars to be a generic/less expensive version of an existing b/tsDMARD, 28 (11%) believed biosimilars to be an identical medication made by a different company, with the remainder [44 (18%)] indicating they were unsure.

**Figure 3.**
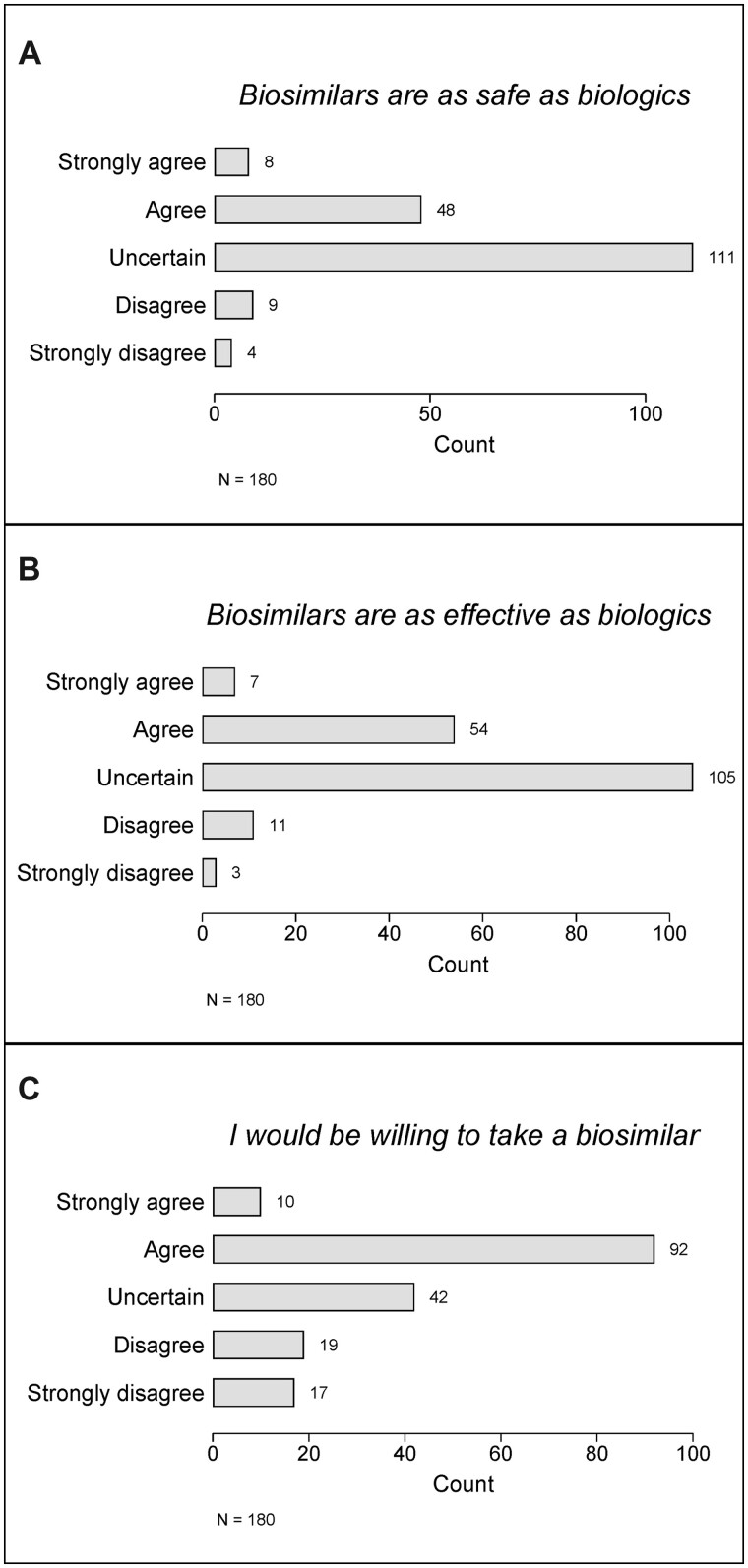
Perceptions of biosimilars in participants familiar with biosimilars (*n* = 180) in terms of (**A**) safety, (**B**) efficacy and (**C**) willingness to take a biosimilar

Of those participants familiar with biosimilars, approximately half [84/180 (47%)], representing 8% of all participants, were aware that biosimilars were available in Australia. There was a great deal of uncertainty among these participants as to whether biosimilars were safe [111/180 (62%); Fig. 3A] and effective [105/180 (58%); Fig. 3B], but a majority also indicated some willingness to take biosimilars [102/180 (57%); Fig. 3C].

**Figure 4.**
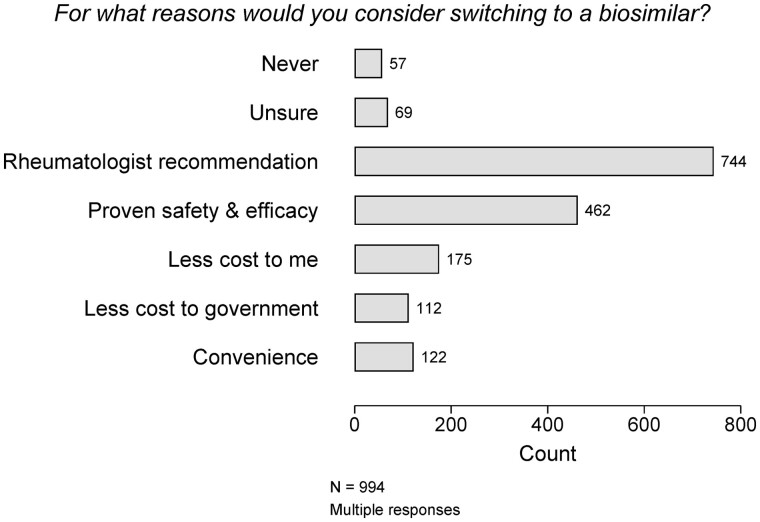
Reasons for considering switching to a biosimilar (all survey participants after review of some biosimilar information)

After reading some supplied information about biosimilars, all survey participants were asked about the circumstances in which they would consider switching to biosimilars (Fig. 4). Only a few participants indicated they were unwilling [57/996 (6%)] to or uncertain about [69/994 (7%)] considering switching. Most participants [744/794 (75%)] indicated they would consider switching to a biosimilar if this was recommended by their rheumatologist. Proven safety and efficacy in clinical trials was also an important consideration for many [462/994 (46%)], with cost and convenience less important.

## Discussion

In this study we surveyed the knowledge and beliefs of Australian patients with inflammatory arthritis regarding b/tsDMARDs and biosimilars. A consistent finding throughout the study, as in previous studies [7, 10, 17, 20], was that patients had a high level of confidence and trust in their rheumatologist to provide information and advice on treatment decisions. Higher levels of trust in the physician, along with active patient participation, has been shown to lead to better patient outcomes in rheumatological diseases [21].

Despite high levels of trust in their rheumatologist’s advice, survey participants currently taking b/tsDMARDs accessed multiple sources for information about their treatment, with most (77%) seeking information from additional sources. This is not unique for b/tsDMARDs and has also been reported in previous studies for methotrexate treatment in RA [7, 17]. Seeking more information, particularly from the internet, was one strategy identified by patients faced with conflicting medication information [6]. Our data suggest that in addition to younger age being a factor in seeking information from multiple sources, medication concerns (as measured by the BMQ) may drive seeking information from sources, such as social media and chat rooms, which are increasingly recognized as platforms for dissemination of misinformation [22].

Substantially less-positive/more-negative information about b/tsDMARDs was a feature of additional b/tsDMARD information sources. It was unclear if the survey participants understood that information from some of these sources was potentially unreliable, but there was considerable uncertainty in their perception of what this information meant. Patients will benefit from improved education about best medication information sources and how to appropriately address conflicting medication information.

Despite differing views obtained from different information sources, survey participants currently taking b/tsDMARDs reported a high level of satisfaction with the information they received and also a strong belief in the necessity of their medication, suggesting they had resolved any conflicting information to their satisfaction.

Only 18% of survey participants expressed familiarity with biosimilars. Even with this lack of familiarity, most participants indicated a willingness to switch to a biosimilar if recommended by their rheumatologist, with only 6% indicating they would never consider it. Only 6% of patients were familiar with biosimilars in a recent, smaller Australian study, but otherwise results were very similar, with 77% of participants open to switching to a biosimilar while only 6% would not consider changing [20]. Awareness of biosimilars was comparatively higher (43%) in a recent study of French patients with rheumatic inflammatory diseases [10], with 15% indicating they would refuse to consider a change. Therefore, familiarity with biosimilars may be comparatively low in Australia despite the Australian government’s Biosimilar Awareness Initiative. Proven efficacy and safety of biosimilars was an important factor in our survey participants, similar to findings in other studies [10, 20]. Cost and convenience were rated as somewhat less important considerations, but these are also likely to be of some consequence when making decision about switching to a biosimilar. Patient bias against generic medications may also be an important factor [20].

The strengths of this study are the good response rate and large number of well-characterized participants with a range of autoimmune inflammatory rheumatic diseases drawn from all over Australia. Potential limitations include whether the participants are representative of the wider range of such patients in Australia and therefore whether the findings are widely generalizable. There is a high rate of b/tsDMARD use in ARAD participants, indicative of moderate–severe disease. Of the patients who participated in this survey, only 2% were found to have low health literacy (as measured by the SILS). Two previous Australian studies that determined health literacy in community rheumatic disease patients found the percentage of patients with low health literacy to be 8.1% and 8.9%, which is higher than in our study population [23, 24]. Therefore the proportion of patients consulting GPs and pharmacists may be higher in the general population. Additionally, as ARAD participants, survey participants are likely to be actively engaged with their disease and its treatment, and technologically literate by virtue of completing an annual ARAD online questionnaire, therefore the percentage of participants consulting online sources may be higher than in the general population. The use of resources such as rheumatology nurses was low (34%), however, this may be more indicative of a relative lack of available nurses compared with rheumatologists than representative of a lack of trust. Our survey was also distributed when few biosimilars were available in Australia, which may account for the low percentage of our population aware of these medications.

## Conclusion

Australian patients with RA, PsA and axSpA have positive attitudes towards b/tsDMARDs overall, although little knowledge of biosimilars specifically. They consult a number of information sources but have a high degree of trust in their rheumatologist with regard to treatment decisions, even if they are unfamiliar with the medication recommended.

## Supplementary Material

rkac099_Supplementary_Data

## Data Availability

Data are not publicly available but interested parties can contact the corresponding author.

## Notes

*Disclosure statement*: The authors have declared no conflicts of interest.
