# Supplementary material for: Attitudes of Australians with inflammatory arthritis to biologic therapy and biosimilars
Source: Rheumatol Adv Pract. 2022 Nov 10;6(3):rkac099. doi: 10.1093/rap/rkac099 (PMC9682816; doi:10.1093/rap/rkac099)
Supplement: rkac099_Supplementary_Data [file rkac099_supplementary_data.zip › Supplementary File 2_Table S1.pdf]

Table S1: Covariate coefficients from Heckman ordinal probit sample selection models for the respective bDMARD information sources utilised, and how favourably that information was perceived

These data were analysed by a Heckman ordinal probit selection model in Stata v16.1. The analyses for each information source were combined, using a seemingly unrelated regression approach (suest) to allow correlation of the standard errors for each individual.

The models for each source have two components:

- The "selection" model component analyses whether a particular source was selected/consulted/asked
- The ordered probit model component analyses how favourable the views were when asked (seven categories: strongly negative, strongly negative, negative, uncertain/neutral, positive, strongly positive, very strongly positive)
- A positive coefficient reflects a positive association between the covariate and whether an information source was utilised, or how favourably that information was perceived. Conversely, a negative coefficient indicates an inverse association

Covariate	Selection Model (source consulted)			Ordered probit model (favourability)		
	Coefficient	Std.Err	p-val	Coefficient	Std.Err	p-val
Rheumatologist						
Age	-0.015	0.016	0.37	-0.008	0.004	0.026
BMQ necessity	0.13	0.22	0.56	0.33	0.07	<0.001
BMQ concerns	-0.21	0.13	0.10	-0.29	0.06	<0.001
Poorer health literacy[1]	0	(omitted)		0.13	0.18	0.47
Post High School education	-0.01	0.35	0.97	0.17	0.09	0.051
Rheumatology Nurse						
Age	-0.001	0.004	0.89	-0.008	0.006	0.20
BMQ necessity	-0.01	0.07	0.91	0.17	0.10	0.089
BMQ concerns	0.08	0.06	0.17	-0.08	0.09	0.36
Poorer health literacy[1]	0.19	0.19	0.32	0.07	0.26	0.78
Post High School education	-0.13	0.10	0.20	0.26	0.14	0.063
GP						
Age	-0.004	0.004	0.35	0.004	0.005	0.49
BMQ necessity	0.06	0.07	0.38	0.25	0.08	0.001
BMQ concerns	0.05	0.06	0.39	-0.09	0.07	0.24
Poorer health literacy[1]	0.41	0.20	0.040	0.32	0.22	0.14
Post High School education	-0.18	0.10	0.063	0.07	0.13	0.60
Pharmacist						
Age	-0.005	0.004	0.23	-0.002	0.005	0.68
BMQ necessity	-0.03	0.07	0.70	0.11	0.10	0.30
BMQ concerns	0.07	0.06	0.22	-0.22	0.08	0.006
Poorer health literacy[1]	0.46	0.19	0.013	0.07	0.18	0.71
Post High School education	-0.11	0.10	0.25	0.05	0.14	0.74
Relatives/Friends						
Age	-0.006	0.004	0.18	-0.013	0.007	0.060
BMQ necessity	0.01	0.08	0.95	0.05	0.11	0.67
BMQ concerns	0.13	0.06	0.040	-0.05	0.09	0.55
Poorer health literacy[1]	0.37	0.19	0.053	0.09	0.24	0.69
Post High School education	-0.11	0.11	0.28	0.02	0.16	0.90
Other patients						
Age	-0.011	0.004	0.016	-0.016	0.006	0.012
BMQ necessity	0.06	0.07	0.39	0.14	0.10	0.17

Covariate	Selection Model (source consulted)			Ordered probit model (favourability)		
	Coefficient	Std.Err	p-val	Coefficient	Std.Err	p-val
BMQ concerns	0.11	0.06	0.082	-0.07	0.11	0.48
Poorer health literacy[1]	0.14	0.19	0.48	-0.25	0.23	0.28
Post High School education	-0.17	0.10	0.11	0.02	0.16	0.92
Educational websites						
Age	-0.014	0.004	0.001	-0.001	0.005	0.84
BMQ necessity	0.08	0.07	0.22	0.04	0.07	0.62
BMQ concerns	-0.01	0.06	0.80	-0.19	0.05	<0.001
Poorer health literacy[1]	-0.09	0.19	0.64	-0.16	0.22	0.46
Post High School education	0.07	0.11	0.56	0.06	0.19	0.75
Other websites						
Age	-0.013	0.004	0.002	-0.017	0.005	0.001
BMQ necessity	-0.02	0.07	0.73	0.06	0.09	0.51
BMQ concerns	0.14	0.06	0.016	-0.19	0.07	0.009
Poorer health literacy[1]	0.02	0.19	0.90	-0.04	0.24	0.88
Post High School education	0.06	0.10	0.53	0.18	0.13	0.17
Social media						
Age	-0.011	0.005	0.015	-0.026	0.007	<0.001
BMQ necessity	0.04	0.08	0.63	-0.03	0.13	0.80
BMQ concerns	0.18	0.07	0.005	0.05	0.11	0.68
Poorer health literacy	0.25	0.20	0.20	-0.02	0.26	0.93
Post High School education	-0.14	0.11	0.19	0.04	0.17	0.83
Chat rooms						
Age	-0.010	0.005	0.034	-0.028	0.007	<0.001
BMQ necessity	0.01	0.08	0.89	-0.05	0.13	0.70
BMQ concerns	0.19	0.07	0.003	0.07	0.11	0.54
Poorer health literacy[1]	0.12	0.20	0.56	-0.22	0.25	0.37
Post High School education	-0.18	0.11	0.098	-0.11	0.17	0.54
Media (other)						
Age	0.003	0.005	0.52	0.001	0.008	0.87
BMQ necessity	0.00	0.08	0.96	-0.08	0.14	0.56
BMQ concerns	0.11	0.07	0.091	-0.02	0.11	0.86
Poorer health literacy[1]	0.20	0.20	0.30	-0.11	0.29	0.71
Post High School education	-0.12	0.11	0.27	0.21	0.17	0.21

[1]Poorer health literacy: Participants were asked a single question as to how often they needed assistance reading health information materials, with answer options ranging from 1 (always) to 5 (never). Scores of 1 (always) or 2 (often) were considered to indicate some difficulty with reading printed health-related material
