# Supplementary material for: Attitudes of Australians with inflammatory arthritis to biologic therapy and biosimilars
Source: Rheumatol Adv Pract. 2022 Nov 10;6(3):rkac099. doi: 10.1093/rap/rkac099 (PMC9682816; doi:10.1093/rap/rkac099)
Supplement: rkac099_Supplementary_Data [file rkac099_supplementary_data.zip › Supplementary File 1_Survey Questions.pdf]

Draft

Patient views regarding biological therapy

1. BIOLOGIC THERAPY

What is a biologic medication?
Biological disease modifying anti-rheumatic drugs (biological DMARDs or bDMARDs) are a group of newer medicines that are used to treat rheumatic diseases (rheumatoid arthritis, ankylosing spondylitis, psoriatic arthritis). These medicines block natural substances called cytokines which contribute to the disease process in rheumatic diseases.

Types
Biologic medication used for arthritis in Australia are listed below:
• Abatacept (Orencia)
• Adalimumab (Humira)
• Certolizumab (Cimzia)
• Etanercept (Enbrel, Brenzys)
• Golimumab (Simponi)
• Infliximab (Remicade)
• Rituximab (Mabthera, Riximyo)
• Secukinumab (Cosentyx)
• Tocilizumab (Actemra)
• Ustekinumab (Stelara)

In addition, there are another group of medications called targeted synthetic DMARDs (tsDMARDs) that have recently come onto the market.
• Baricitinib (Olumiant)
• Tofacitinib (Xeljanz)

1.1 I am currently taking a biologic or tsDMARD? ☐ Yes ☐ No

If YES → **1.1.1** Which?

☐ Abatacept (Orencia) ☐ Golimumab (Simponi) ☐ Tocilizumab (Actemra)
☐ Adalimumab (Humira) ☐ Infliximab (Remicade) ☐ Ustekinumab (Stelara)
☐ Certolizumab (Cimzia) ☐ Rituximab (Mabthera, Riximyo) ☐ Baricitinib (Olumiant)
☐ Etanercept (Enbrel, Brenzys) ☐ Secukinumab (Cosentyx) ☐ Tofacitinib (Xeljanz)

1.2 I used to take a biologic or tsDMARD but it was stopped? ☐ Yes ☐ No

If YES → **1.2.1** Which?

☐ Abatacept (Orencia) ☐ Golimumab (Simponi) ☐ Tocilizumab (Actemra)
☐ Adalimumab (Humira) ☐ Infliximab (Remicade) ☐ Ustekinumab (Stelara)
☐ Certolizumab (Cimzia) ☐ Rituximab (Mabthera, Riximyo) ☐ Baricitinib (Olumiant)
☐ Etanercept (Enbrel, Brenzys) ☐ Secukinumab (Cosentyx) ☐ Tofacitinib (Xeljanz)

1.2.2 Specify reason for stopping

If 1.1 is No

1.3 I am not on a biologic or tsDMARD because

☐ I used to take a biologic but it was stopped
☐ I was offered a biologic but I decided not to take it, please specify
☐ I was not offered a biologic by my doctor
☐ I have never heard of biologic medications

If 1.3 is answered Go to 3. Biosimilars

1. BIOLOGIC THERAPY (if currently taking biologic)

1.4 Where did you get your advice and formation about your biologic or tsDMARD medication from AND was this advice positive? (please tick all that apply)

	Not asked	Very Strongly Positive	Strongly Positive	Positive	Uncertain / Neutral	Negative	Strongly Negative	Very Strongly Negative
a) Rheumatologist	☐	☐	☐	☐	☐	☐	☐	☐
b) Rheumatology nurse	☐	☐	☐	☐	☐	☐	☐	☐
c) General Practitioner	☐	☐	☐	☐	☐	☐	☐	☐
d) Pharmacist	☐	☐	☐	☐	☐	☐	☐	☐
e) Relative/Friends	☐	☐	☐	☐	☐	☐	☐	☐
f) Other patients	☐	☐	☐	☐	☐	☐	☐	☐
Internet Educational websites (eg Australian Rheumatology Association / Arthritis Australia)	☐	☐	☐	☐	☐	☐	☐	☐
h) Other internet websites (Google, Wikipedia, etc)	☐	☐	☐	☐	☐	☐	☐	☐
i) Social media (Facebook, Twitter, Instagram)	☐	☐	☐	☐	☐	☐	☐	☐
j) Internet chat rooms/forums	☐	☐	☐	☐	☐	☐	☐	☐
k) Media (newspapers, magazines, television, radio)	☐	☐	☐	☐	☐	☐	☐	☐

1.5 How satisfied are you with the information you have received about your biologic or tsDMARD medication?

Not satisfied at all ____________________ Very satisfied

1.6 If the effectiveness of all biologic or tsDMARD medications were equal, the most important factor influencing my choice of biologic or tsDMARD would be:

- ☐ Cost of the medication to me
- ☐ How it was given (injection under the skin that I give myself/intravenous drip/tablet)
- ☐ Ease of access to the medication
- ☐ Recommendation of my rheumatologist
- ☐ Recommendation of my pharmacist
- ☐ Recommendation of my rheumatology nurse
- ☐ Recommendation by close friends/family
- ☐ Information obtained online or via media
- ☐ Other, please specify ____________________

1.7 Comments

____

2. YOUR VIEWS ABOUT BIOLOGICS (if currently taking biologic)

2.1 We you like to ask you about your personal views about BIOLOGICS prescribed for your RHEUMATIC DISEASE.

These are statements other people have made about their medicines.

Please show how much you agree or disagree with them by ticking the appropriate box.

Views about BIOLOGICS or tsDMARDs prescrbed for you	Strongly Agree	Agree	Uncertain	Disagree	Strongly Disagree
a) My health, at present, depends on my biologic or tsdMARD	☐	☐	☐	☐	☐
b) My life would be impossible without my biologic or tsDMARD	☐	☐	☐	☐	☐
c) Without my biologic or tsDMARD I would be very ill	☐	☐	☐	☐	☐
d) My health in thet future will depend on my biologic or tsDMARD	☐	☐	☐	☐	☐
e) My biologic or tsDMARD protects me from becoming worse	☐	☐	☐	☐	☐
f) Having to take a biologic or tsDMARD worries me	☐	☐	☐	☐	☐
g) My biologic or tsDMARD is a mystery to me	☐	☐	☐	☐	☐
h) My biologic or tsDMARD disrupts my life	☐	☐	☐	☐	☐
i) I sometimes worry about becoming too dependent on my biologic or tsDMARD	☐	☐	☐	☐	☐
j) I sometimes worry about the long term effects of my biologic or tsDMARD	☐	☐	☐	☐	☐

2.2 Comments

2.3 Views about BIOLOGIC or tsDMARD prescrbed for you COMPARED to DMARDS.

(The most common DMARDs are methotrexate, sulfasalazine, hydroxyxhloroquine, and leflunomide. Less frequently used medications include gold, azathioprine, and cyclosporin)

	Strongly Agree	Agree	Uncertain	Disagree	Strongly Disagree
a) Biologic or tsDMARD medications have less side effects than other DMARDs	☐	☐	☐	☐	☐
b) Biologic or tsDMARD medications are stronger than other DMARDs	☐	☐	☐	☐	☐
c) Biologic or tsDMARD medication supress the immune system more than other DMARDs	☐	☐	☐	☐	☐
d) Biologic or tsDMARD medications are more toxic than other DMARDs	☐	☐	☐	☐	☐
e) Biologic or tsDMARD medications make me more likely to get infections than other DMARDs	☐	☐	☐	☐	☐

2.4 Comments

3. BIOSIMILARS

3.1 Have you ever heard of the term 'biosimilar'? ☐ Yes ☐ No ☐ Unsure

If No → Go to 4. What is a biosimilar

If YES or Unsure → **3.1.1** What does the term 'biosimilar medicine' (biosimilar) mean?

☐ Not sure

☐ A biologic medication identical to an existent (originator) but made by a different company

☐ A generic version of an existent (originator) biologic which may be less expensive

☐ A close copy of an existent (originator) biologic which has been shown to have no significant difference to the originator biologic

3.1.2 Biosimilars are already available for treatment of rheumatic diseases in Australia? ☐ Yes ☐ No ☐ Not sure

3.1.3 How much do you agree with the statements below about biosimilars?

	Strongly Agree	Agree	Uncertain	Disagree	Strongly Disagree
a) Biosimilars are as safe as biologics	☐	☐	☐	☐	☐
b) Biosimilars are as effective as biologics	☐	☐	☐	☐	☐
c) I would be willing to take a biosimilar if my physician suggests it	☐	☐	☐	☐	☐

3.1.4 What more information do you need to decide if you would take a biosimilar? ________

3.1.5 I am currently prescribed a biosimilar by my rheumatologist ☐ Yes ☐ No ☐ Not sure

If YES → **3.1.5.1** Which one? ________

4. WHAT IS A BIOSIMILAR?

Biological disease modifying anti-rheumatic drugs (biological DMARDs or bDMARDs) are a group of newer medicines that are used to treat rheumatic diseases (rheumatoid arthritis, ankylosing spondylitis, psoriatic arthritis). These medicines block natural substances called cytokines which contribute to the disease process in rheumatic diseases.

These medicines are derived from or produced by bacteria, yeasts, cells, plants or animals and block natural substances called cytokines which contribute to the disease process in rheumatic diseases. There are multiple different types of biological DMARDs and the first of each type is called an 'originator'.

For many years, generic medicines have been available as less expensive alternatives to traditional brands.

Unlike generic medicines, it is impossible to make an exact copy of a biologic medicine due to the manufacturing process. However, it is possible to make a biologic that is highly similar to a biologic product and this is referred to as a "biosimilar". The term "biosimilar" refers to a biologic product which has been developed and tested to ensure that there is no significant difference between the "biosimilar" and the original biologic.

4.1 Do you use generic brands of other medicines (such as paracetamol, aspirin, ibuprofen, attorvastatin)? ☐ Yes ☐ No ☐ Not sure

4.2 Comments ________

4. WHAT IS A BIOSIMILAR?

4.3 What do you feel are the main differences between biologics (originators) and biosimilars?
You may choose more than one answer)

☐ Quality ☐ Efficacy ☐ Safety ☐ Price ☐ None of the above

4.4 When the price of the biologic (originator) is the same as the biosimilar, which one would you prefer to be treated with?

☐ Biologic (originator)

☐ Biosimilar

☐ Whichever my rheumatologist through would be best for my condition

☐ Not sure

4.5 When the price to the government of the biologic (originator) is more than the biosimilar, which one would you prefer to be treated with?

☐ Biologic (originator)

☐ Biosimilar

☐ Whichever my rheumatologist through would be best for my condition

☐ Depends on the price difference

☐ Not sure

4.6 If you were/are on treatment with a biologic (originator) when would you consider switching to a biosimilar? (you may choose multiple answers)

☐ Never

☐ If my rheumatologist recommended it

☐ When the clinical trial had proven that the safety and effectiveness of the biosimilar was equal to the biologic (originator) for my disease

☐ When the cost to me was less than the biologic (originator)

☐ When the cost to the government was less than the biologic (originator)

☐ If thebiosimilar was more convenient for me

☐ Unsure

4.7 Comments

5. OTHER MEDICATIONS

We are intered in the other arthritis medications that you are on

5.1 Methotrexate

☐ I am currently taking methotrexate → **5.1.1** Dose ☐ **5.1.2** Adminstered ☐ Oral/Tablet ☐ Injection

☐ I used to take methotrexate but it was stopped → **5.1.3** Reason
- ☐ Didn't work
- ☐ Side effects
- ☐ Didn't want to take it anymore
- ☐ Other → **5.1.3.1** Specify ☐

☐ It was offered to me but I decided not to take it → **5.1.4** Reason ☐

☐ None of the above

5.2 Leflunomide

☐ I am currently taking leflunomide → **5.2.1** Dose ☐ 10mg ☐ 20mg ☐ Other **5.2.1.1** Specify ☐

☐ I used to take leflunomide but it was stopped → **5.2.2** Reason
- ☐ Didn't work
- ☐ Side effects
- ☐ Didn't want to take it anymore
- ☐ Other → **5.2.2.1** Specify ☐

☐ It was offered to me but I decided not to take it → **5.2.3** Reason ☐

☐ None of the above

5.3 Prednisolone

☐ I am currently taking prednisolone → **5.3.1** Daily Dose ☐

5.3.2 How many days per week do you take it ☐

5.3.3 What year were you started on prednisolone ☐

☐ I used to take prednisolone but it was stopped → **5.3.4** Reason
- ☐ Didn't work
- ☐ Side effects
- ☐ Didn't want to take it anymore
- ☐ Other → **5.3.4.1** Specify ☐

☐ It was offered to me but I decided not to take it → **5.3.5** Reason ☐

☐ None of the above

5.4 How ofter do you need to have someone help you when you read instructions, pamphlets, or other written material from your doctor or pharmacy?

☐ Always ☐ Often ☐ Sometimes ☐ Rarely ☐ Never
